# Tissue distribution and elimination after oral and intravenous administration of different titanium dioxide nanoparticles in rats

**DOI:** 10.1186/1743-8977-11-30

**Published:** 2014-07-03

**Authors:** Liesbeth Geraets, Agnes G Oomen, Petra Krystek, Nicklas R Jacobsen, Håkan Wallin, Michel Laurentie, Henny W Verharen, Esther FA Brandon, Wim H de Jong

**Affiliations:** 1 National Institute for Public Health and the Environment (RIVM), PO Box 1, 3720 BA, Bilthoven, The Netherlands; 2 Philips Innovation Services, High Tech Campus 11, 5656 AE Eindhoven, The Netherlands; 3 National Research Centre for the Working Environment (NRCWE), Lersø Parkallé 105, Copenhagen DK-2100, Denmark; 4 French Agency for Food, Environmental and Occupational Health and Safety (ANSES), Fougères Laboratory, 10B rue Claude Bourgelat, Javené CS 40608 35306 Fougères Cedex, France

**Keywords:** Titanium dioxide, Nanoparticles, Kinetics, Tissue distribution

## Abstract

**Objective:**

The aim of this study was to obtain kinetic data that can be used in human risk assessment of titanium dioxide nanomaterials.

**Methods:**

Tissue distribution and blood kinetics of various titanium dioxide nanoparticles (NM-100, NM-101, NM-102, NM-103, and NM-104), which differ with respect to primary particle size, crystalline form and hydrophobicity, were investigated in rats up to 90 days post-exposure after oral and intravenous administration of a single or five repeated doses.

**Results:**

For the oral study, liver, spleen and mesenteric lymph nodes were selected as target tissues for titanium (Ti) analysis. Ti-levels in liver and spleen were above the detection limit only in some rats. Titanium could be detected at low levels in mesenteric lymph nodes. These results indicate that some minor absorption occurs in the gastrointestinal tract, but to a very limited extent.

Both after single and repeated intravenous (IV) exposure, titanium rapidly distributed from the systemic circulation to all tissues evaluated (i.e. liver, spleen, kidney, lung, heart, brain, thymus, reproductive organs). Liver was identified as the main target tissue, followed by spleen and lung. Total recovery (expressed as % of nominal dose) for all four tested nanomaterials measured 24 h after single or repeated exposure ranged from 64-95% or 59-108% for male or female animals, respectively. During the 90 days post-exposure period, some decrease in Ti-levels was observed (mainly for NM-100 and NM-102) with a maximum relative decrease of 26%. This was also confirmed by the results of the kinetic analysis which revealed that for each of the investigated tissues the half-lifes were considerable (range 28–650 days, depending on the TiO_2_-particle and tissue investigated). Minor differences in kinetic profile were observed between the various particles, though these could not be clearly related to differences in primary particle size or hydrophobicity. Some indications were observed for an effect of crystalline form (anatase vs. rutile) on total Ti recovery.

**Conclusion:**

Overall, the results of the present oral and IV study indicates very low oral bioavailability and slow tissue elimination. Limited uptake in combination with slow elimination might result in the long run in potential tissue accumulation.

## Background

Titanium dioxide (TiO_2_) is primarily used as a whitening agent due to its brightness and resistance to discoloration in consumer products and food. Titanium dioxide is approved as a white-colored food additive in Europe (E171) [1]. Examples of foods with a high content of titanium dioxide are chewing gums and candies. Personal care products like toothpastes, lip balms, shampoos, deodorants and sunscreens often contain titanium dioxide as ingredient [2,3]. Analysis by transmission electron microscopy (TEM) of one single batch of food-grade E171 titanium dioxide by Weir *et al*. showed that 36% of the particles (number-based) have a particle size smaller than 100 nm [2]. Using scanning electron microscopy (SEM)-analysis of 7 food-grade E171 titanium dioxide types, Peters *et al*. reported that about 10% of the particles (number-based) have a size smaller than 100 nm [4]. It should be noted that both according to the data of Weir et al. [2] and Peters et al. [4], E171 would not be considered as a nanomaterial according to the EU Recommendation [5]. In addition, the nano-form of titanium dioxide is recently also applied in paint as an antimicrobial agent due to its hydroxyl radical generative property [6]. In some products such as sun screens titanium dioxide can be mainly applied as nanoparticles, whereas in many other products in which titanium dioxide is applied as whitening agent only fractions of the particles are of nanosize [2,4,7].

For human risk assessment purposes, information on the kinetics of titanium dioxide in the human body is essential. This is particularly the case for chemicals with a potency to accumulate with normal use. The overall resultant of absorption, distribution, metabolism and excretion (ADME), i.e. internal exposure, will determine target tissue doses upon human exposure to chemicals and this internal exposure will be critical for the ultimate systemic adverse health effects. In case of no absorption, the potential risk upon exposure is expected to be limited to local effects at the exposure site. Information on kinetics, particularly internal exposure in time, facilitates comparison between toxicity studies with typical human situations.

Human exposure to titanium dioxide nanoparticles can occur via various routes. For consumers, based on potential external exposure via consumer products, the oral and dermal routes are considered most relevant. Oral exposure may occur as regularly consumed food products contain E171. Although the additive E171 cannot be considered as a nanomaterial according to the current recommendation for a nanodefinition, exposure and uptake of the nanofraction of E171 cannot be excluded. In addition, oral exposure to consumer products such as lip balm containing titanium dioxide and swallowing of (nano)particles after inhalation exposure and transport via the muco-ciliary escalator to the mouth, might contribute to the total oral exposure. Information on the extent of oral absorption of these particles provides information on whether internal exposure is possible. Furthermore, information on the kinetic profile, i.e. what are the target tissues, and to which extent does elimination from the target tissues occur, provides insight in the potential to accumulate in specific tissues in daily use.

In general, nanomaterials rapidly distribute from blood to tissues [8-11]. Particularly, highly perfused reticulo-endothelial system (RES)-containing tissues such as liver, and spleen are target tissues for nanomaterials. Distribution across protecting membranes has also been observed as nanoparticles have been detected, though in low quantities, in brain, fetus and testis, whereas the presence of nanoparticles in the brain might also occur after inhalation via the olfactory route [12]. Furthermore, elimination is in general quite slow, and for metal and metal oxide nanoparticles like titanium dioxide metabolic degradation does not seem to occur. Elimination of these materials may rather be related to dissolution, which can be very slow. Limited elimination in combination with a low fraction that becomes systemically available will eventually result in accumulation in tissues upon repeated exposure.

Some knowledge on the kinetics or tissue distribution of titanium dioxide nanoparticles upon exposure via various routes (oral, intravenous, dermal and inhalation) is already available [13,14]. However, information for oral absorption and the elimination from tissues following longer time periods are limited. In other studies, tissue distribution after exposure via the various routes was found to be mostly to liver, kidney, spleen and lungs [15-24]. Dermal absorption of nano- or micro- titanium dioxide *in vivo* (humans, animals) or *in vitro* (porcine or human skin) is very low, mostly resulting in values below the detection level [24-28]. One study reported systemic bioavailability after dermal exposure [23]. However, doubts were expressed on the results because of deficiencies in the (reported) characterization, high values of Ti in the control group and most importantly the study design could not exclude oral intake [29].

Absorption of titanium dioxide showed differences among the various exposure routes. Recently, results of an oral 13-week repeated dose study showed low oral absorption of titanium dioxide nanoparticles (based on limited increased blood and tissue titanium levels; not further quantified) [30]. Furthermore, there is little knowledge on the differences in kinetics between different titanium dioxide nanomaterials and how this relates to physicochemical characteristics such as size.

The aim of this study was to obtain better kinetic input for human risk assessment of titanium dioxide nanomaterials. Titanium dioxide particles with different size-range, crystalline form (anatase and rutile as most commonly used) and hydrophobicity were investigated in a single and repeated (5x) dose study in rats. These materials are all commercially manufactured and available via the JRC repository (EU Joint Research Centre (JRC), Ispra, Italy). Both intravenous and oral administration routes were investigated. The oral route was applied as this was considered a relevant exposure route for consumers/general population. The intravenous route was included to obtain insight into the tissue distribution and elimination profile of titanium dioxide particles under conditions of 100% systemic bioavailability, which also makes it possible to obtain a reliable concentration-time relation of Ti in blood and multiple tissues. Dose levels that are considered realistic and relevant for human exposure were applied in our study. Finally, based on general kinetic behavior of nanomaterials and the relevance for risk assessment, emphasis was furthermore put on the potential long elimination period and therefore a 90-day recovery period was included in the present study.

## Results

### Characteristics of titanium dioxide test materials

#### Oral study

The number size distribution of the suspended TiO_2_ particle preparations used for oral gavage was characterized by dynamic light scattering (DLS).

**Figure 1:**
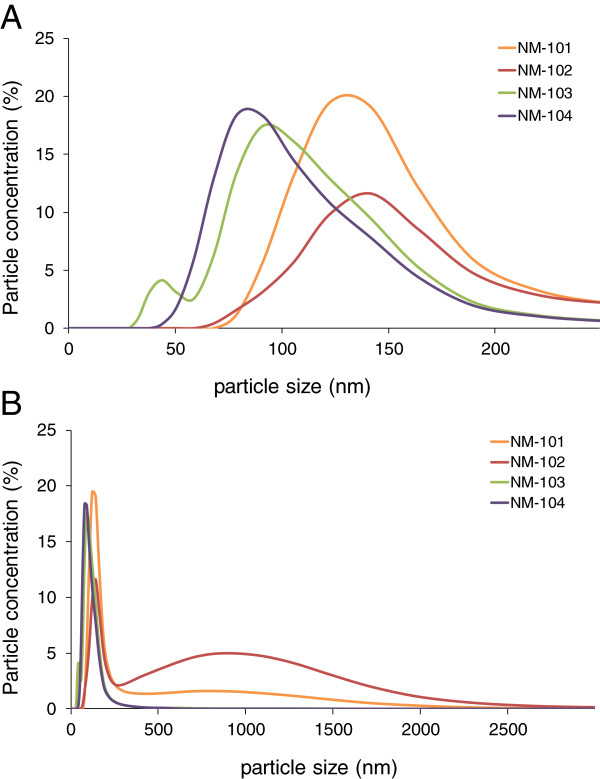
***Oral study: *Hydrodynamic number size distribution of NM-101/NM-102/NM-103/NM-104 of the suspended TiO**_**2 **_**particle preparations used for oral gavage (A: results for particle size 0–250 nm; B: results for particle size 0–3000 nm).** The majority of particles were observed as agglomerates/aggregates between 50–200 nm. Suspensions of NM-101 and NM-102 contained an additional mode between 250 and 2500 nm. No agglomerates/aggregates were observed above 3 μm. Results are the mean of 12 analyses conducted on two separate days.

All TiO_2_ containing suspensions gave reliable results when analyzed according to the DTS software (Table 1). The majority of particles occurred as agglomerates/aggregates measuring between 80–150 nm (peak number distributions, Figure 1). Suspensions of NM-103, and NM-104 contained the smallest agglomerates/aggregates (80–90 nm) whereas the largest were observed with NM-101 and NM-102 (140–150 nm). NM-101 and NM-102 additionally showed a bi-modal size-distribution with another much less frequent mode of ~1 μm. No particles were observed between 3 μm and 10 μm (the upper limit of the Zetasizer nano ZS) for any of the materials.

**Table 1:** **Z-average (intensity distribution peak) and polydispersity index (PI) of the TiO**_**2**_**suspensions used for oral gavage**

**Material**	**Concentration (mg/mL)**	**Vehicle**	**Z-Average (intensity; nm)**	**Pl**
NM-101	2.56	EtOH/RSA/PBS	278.49 ± 7.7	0.32 ± 0.03
NM-102	2.56	EtOH/RSA/PBS	366.83 ± 13.64	0.29 ± 0.00
NM-103	2.56	EtOH/RSA/PBS	127.18 ± 0.42	0.38 ± 0.01
NM-104	2.56	EtOH/RSA/PBS	107.73 ± 3.85	0.27 ± 0.00
control	2.56	EtOH/RSA/PBS	unreliable results*	0.82

*due to the high PI.

Results are the mean ± SD of 12 analyses conducted on two separate days.

#### IV study

**Figure 2:**
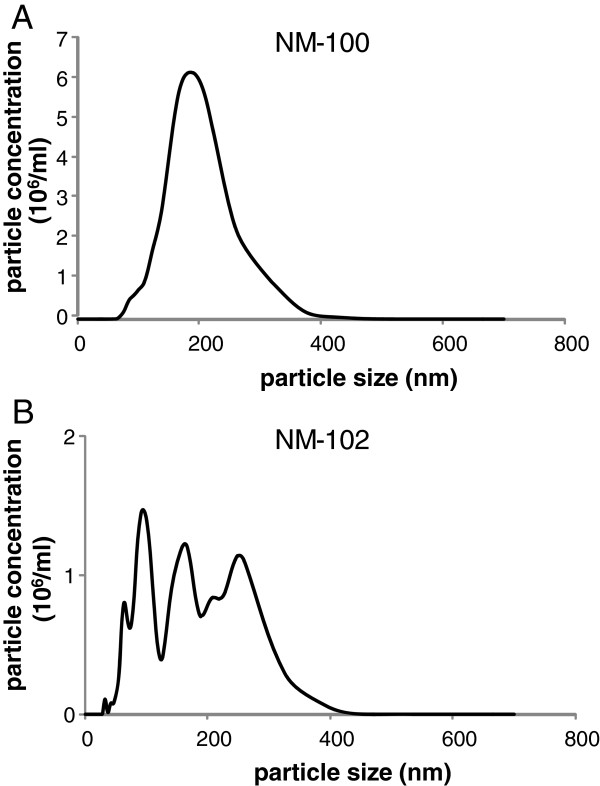
***IV study: *Hydrodynamic particle number size distribution of (A) NM-100 and (B) NM-102.** Analysis was performed with the NanoSight particle tracking equipment. Mean of 3 independent measurements.

Particle tracking with the NanoSight equipment was performed for NM-100, NM-102, NM- 103 and NM-104 used for the IV studies. The evaluation was performed just after the dispersions were prepared. Representative figures are presented for NM-100 and NM-102 (Figures 2A and B, respectively). For NM-100 one single peak was observed at the nominal size of the particle diameter (200–220 nm). For NM-102 different peaks could be identified indicating the presence of polydispersity of the initial sample (multiple sizes) or the occurrence of agglomeration/aggregation of the individual nanoparticles into larger agglomerates/aggregates of nanoparticles. Agglomerates/aggregates of nanoparticles with a size of approximately 60 nm, 100 nm, 160 nm, and 250 nm could be identified by the observed peaks.

### Tissue distribution of titanium dioxide particles after oral dosing

For the oral administration liver, spleen and mesenteric lymph nodes were selected as priority target tissues. The tissue distribution after oral exposure to NM-101, NM-102, NM-103, and NM-104 was evaluated for the repeated dose schedule only. All liver and spleen tissue samples from rats (NM-101: male and female rats, NM-102, NM-103 and NM-104: male rats) exposed five times contained very low titanium levels, with levels mostly below the limit of detection of 0.03 μg/g (Table 2). In exposed animals, twenty-eight measurements were below the LOD (0.03 μg/g tissue), 1 was at the LOD (NM-102 liver) and 1 was above the LOD (male liver NM-103). In control animals twice a measurement at the LOD was observed, while 10 times the level was below the LOD.

**Table 2:** **Ti-levels (**μ**g Ti/g tissue) in liver and spleen tissue samples of orally-treated rats**

			**Liver**			**Spleen**		
	**Dose (mg)**		**Animal 1**	**Animal 2**	**Animal 3**	**Animal 1**	**Animal 2**	**Animal 3**
Control	5x 0	♂	< 0.03	< 0.03	< 0.03	< 0.03	< 0.03	< 0.03
NM-101	5x 2.304	♂	< 0.03	< 0.03	< 0.03	< 0.03	< 0.03	< 0.03
NM-102	5x 2.304	♂	< 0.03	**0.03**	< 0.03	< 0.03	< 0.03	< 0.03
NM-103	5x 2.304	♂	**0.08**	< 0.03	< 0.03	< 0.03	< 0.03	< 0.03
NM-104	5x 2.304	♂	< 0.03	< 0.03	< 0.03	< 0.03	< 0.03	< 0.03
Control	5x 0	♀	< 0.03	< 0.03	**0.03**	< 0.03	< 0.03	**0.03**
NM-101	5x 2.304	♀	< 0.03	< 0.03	< 0.03	< 0.03	< 0.03	< 0.03

**Figure 3:**
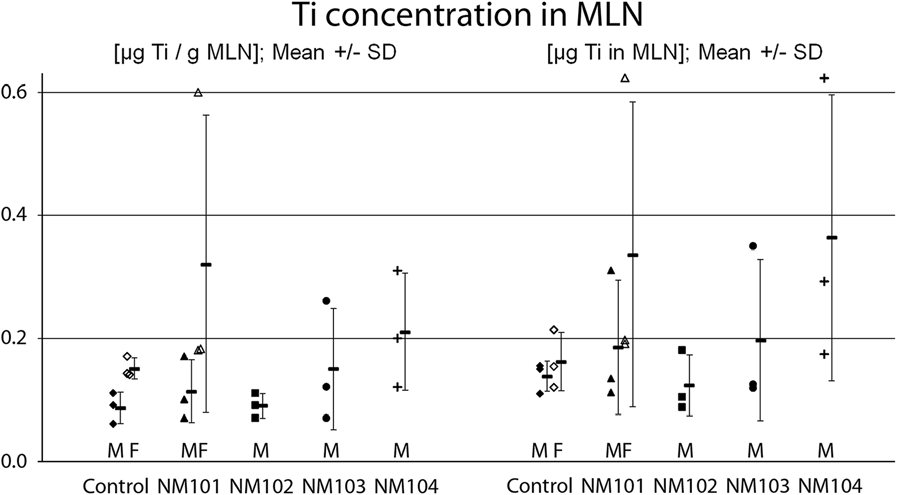
***Oral study: *Determination of Ti concentration in gut mesenteria with mesenteric lymph nodes (designated MLN) from rats exposed via the oral route on 5 consecutive days for either control or TiO2-suspensions.** Ti-concentrations are illustrated per gram MLN (left) or for the whole MLN tissue (right). M, Males and F, Females.

All samples of MLN tissue contained amounts above the limit of detection (Figure 3). Lowest detected concentration was 0.07 μg Ti/g MLN (left panel) or 0.11 μg Ti for the whole MLN (right panel), well above the LOD. Titanium levels in MLN of TiO_2_ exposed animals were, except for NM-104, similar to control animals. It appears that female rats (control and NM-101) have a slightly higher Ti concentration in MLN determined per g/MLN, probably due to a lower weight of MLN and higher relative dosing of TiO_2_. MLN of females and males weighed on average 1.08 g and 1.56 g, respectively. This means that μg Ti are similar when mentioned/g or for the whole MLN for females.

In view of the low levels measured after the repeated administration (Table 2), organs of the single dose schedule were not analysed.

The results indicate that after oral administration absorption of TiO_2_ is very low.

### Tissue distribution of titanium dioxide nanoparticles after IV administration

Data of male and female animals will be presented and described separately in this paragraph, though the focus will be on the male animals. In general, female animals showed a similar kinetic profile as male animals. A supplementary file is provided containing a full overview of the titanium data of female animals (i.e. titanium levels expressed as μg/g tissue).

**Figure 4:**
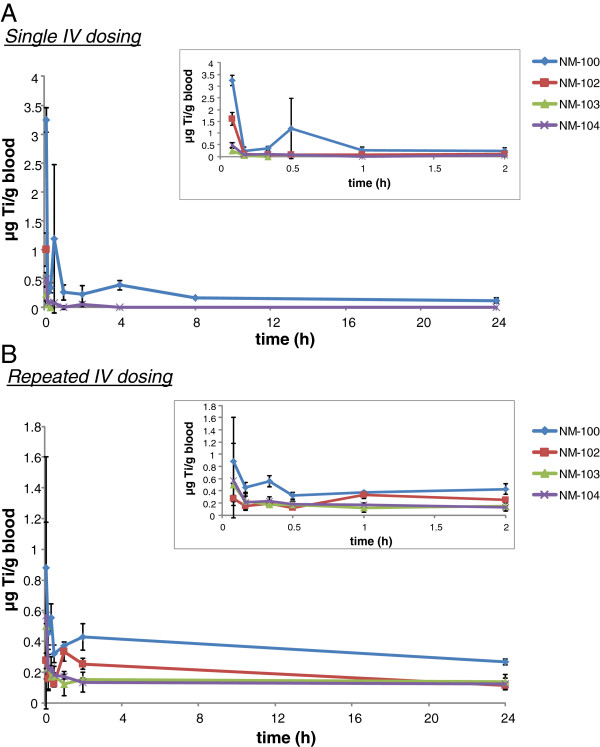
***IV study: *** **Blood kinetics of Ti (μg Ti/g blood) after single IV (A) and repeated IV (B) dosing with NM-100, NM-102, NM-103 and NM-104 in male rats. Insert: blood kinetics during the first two hours.**

**Figure 5:**
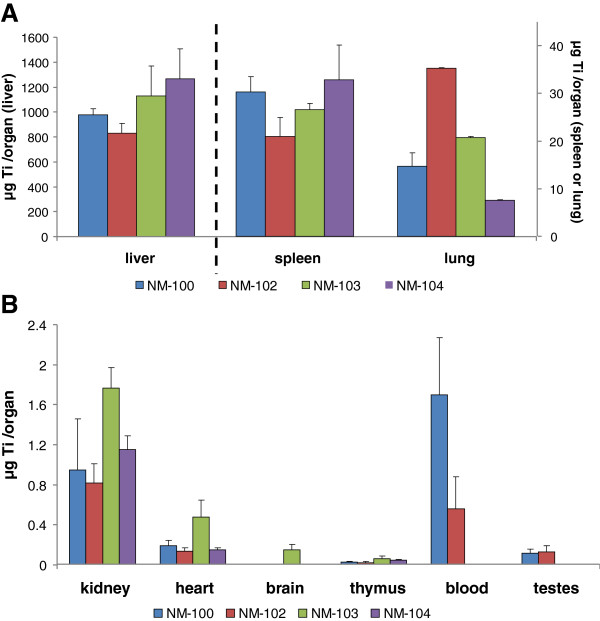
***IV study: *Organ distribution (μg/organ) of Ti on Day 2 after single IV dosing with NM-100, NM-102, NM-103 and NM-104 in male rats (LOD: 0.05** **μg/g). A**: liver, spleen and lungs, **B**: kidney, heart, brain, thymus, blood and testis. NM-100: brain < LOD. NM-102: brain < LOD. NM-103: blood and testis < LOD. NM-104: brain, blood and testis < LOD.

**Figure 6:**
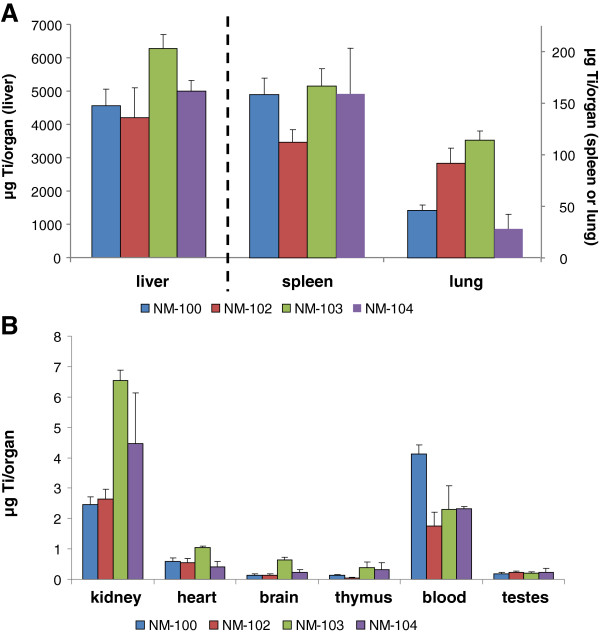
***IV study: *Organ distribution (μg/organ) of Ti on Day 6 after repeated IV dosing with NM-100, NM-102, NM-103 and NM-104 in male rats (LOD: 0.05** **μg/g). A**: liver, spleen and lungs, **B**: kidney, heart, brain, thymus, blood and testis.

**Figure 7:**
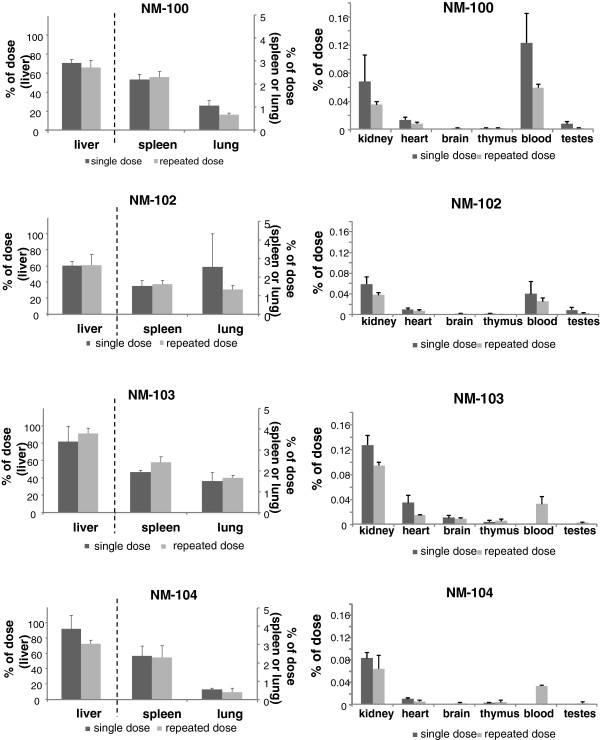
***IV study: *Organ distribution of Ti as percentage of administered dose at 24 h after the last dosing after single (i.e. Day 2) and repeated (i.e. Day 6) IV dosing with NM-100, NM-102, NM-103, and NM-104 in male rats.** (LOD: 0.05 μg/g). left: liver, spleen and lung, right: kidney, heart, brain, thymus, blood and testes. NM-100: brain (single exposure) < LOD. NM-102: brain (single exposure) < LOD. NM-103: blood and testis (all single exposure) < LOD. NM-104: brain, blood and testis (all single exposure) < LOD.

Titanium levels in control (vehicle-exposed) animals were near and below the detection limits in all investigated tissues.The blood kinetics of IV-administered titanium dioxide (single and repeated exposure) in male rats are presented in Figures 4A and B. Analysis of titanium in blood revealed a rapid decline of titanium levels. After the initial rapid decline (within <0.5 hour), the plasma titanium levels remained constant with only a small decrease during post-exposure periods of up to 14 days (data not shown).After single administration, titanium could be detected at the first tissue measurement point (i.e. 24 hours after administration) in all investigated tissues, at levels above those as measured in control (vehicle-treated) animals (Figure 5, male animals). Repeated exposure to all titanium dioxide nanomaterials was found to result in up to 5 times higher tissue titanium levels (expressed as μg/tissue) compared to single dose, indicating that during this time frame no significant elimination occurred and a dose proportional accumulation occurred (Figure 5 and Figure 6, male animals). This also becomes apparent when expressing the titanium levels as % of the dose administered, no clear differences in titanium-levels were observed at 24 hours after the last dosing (Figure 7 (male) or 8 (female) single vs. repeated exposure).

**Figure 8:**
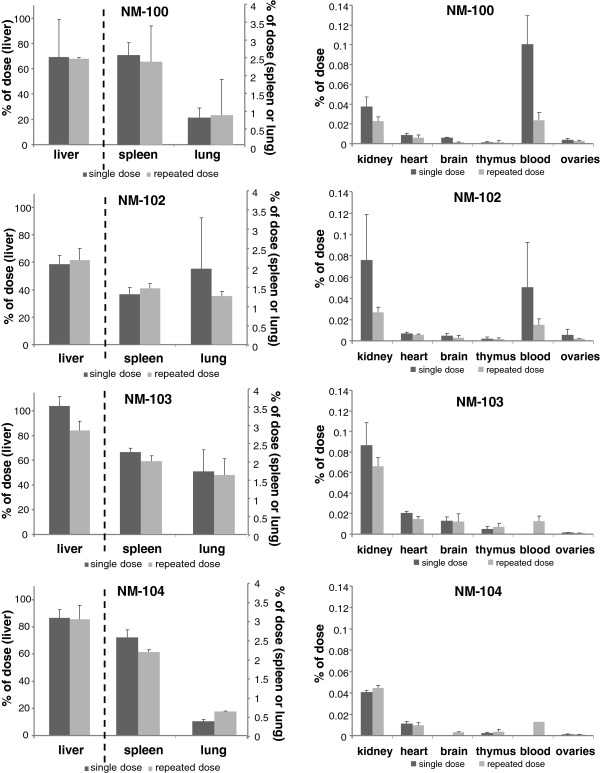
***IV study: *Organ distribution of Ti as percentage of administered dose at 24 h after the last dosing after single (i.e. Day 2) and repeated (i.e. Day 6) IV dosing with NM-100 (A), NM-102 (B), NM-103 (C), and NM-104 (D) in female rats.** (LOD: 0.05 μg/g). Left: liver, spleen and lung, Right: kidney, heart, brain, thymus, blood and ovaria. NM-103: blood (single exposure) < LOD. NM-104: blood and brain (single exposure) < LOD.

**Figure 9:**
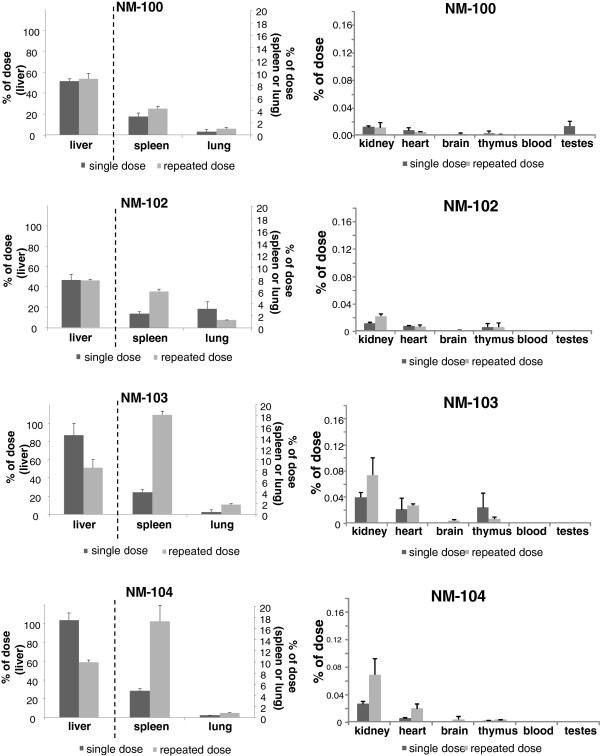
***IV study: *Organ distribution of Ti as percentage of administered dose on Day 90 after single and repeated IV dosing with NM-100 (A), NM-102 (B), NM-103 (C), and NM-104 (D) in male rats.** (LOD: 0.05 μg/g). Left: liver, spleen and lung, Right: kidney, heart, brain, thymus, blood and testes. NM-100: brain (single), blood (single + repeated), testis (repeated) < LOD. NM-102: brain (single), blood (single + repeated), testis (single + repeated) < LOD. NM-103: brain (single), testis (single + repeated) < LOD, blood (single + repeated): not measured. NM-104: brain (single), testis (single + repeated) < LOD, blood (single + repeated): not measured.

**Figure 10:**
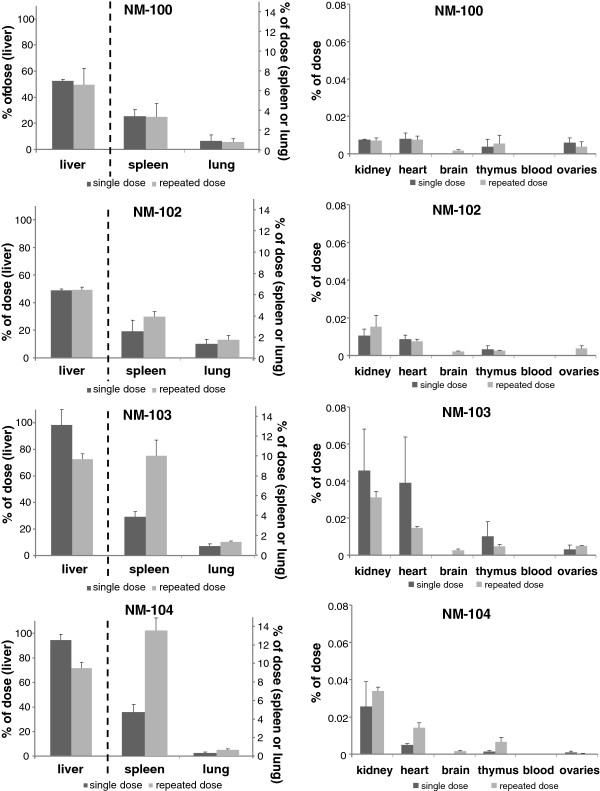
***IV study: *Organ distribution of Ti as percentage of administered dose on Day 90 after single and repeated IV dosing with NM-100 (A), NM-102 (B), NM-103 (C), and NM-104 (D) in female rats.** (LOD: 0.05 μg/g). Left: liver, spleen and lung, Right: kidney, heart, brain, thymus, blood and ovaria. NM-100: brain (single exposure) < LOD, blood (single + repeated) < LOD. NM-102: brain + ovaries (single exposure) < LOD, blood (single + repeated) < LOD. NM-103: brain (single exposure) < LOD, blood (single + repeated): not measured. NM-104: brain (single exposure) < LOD, blood (single + repeated): not measured.

Figure 7 and Figure 8 show the recovery for each investigated tissue as percentage of the dose administered at 24 h after the last administration for male and female animals respectively. After single dosing, the highest tissue distribution in males was observed (in descending order) in liver, spleen and lung for all titanium dioxide nanomaterials, with recovery per tissue of 60-92%, 1.5-2.4% and 0.5-2.6% respectively, at 24 hours after dosing. Other tissues contained less than 0.3% of the dose administered. In female animals, highest titanium levels were also found in liver, spleen and lung tissue for all titanium dioxide nanomaterials, with recovery per tissue of 59-104%, 1.3-2.6% and 0.4-2.0%, respectively. Also after repeated dosing, highest tissue levels were found in liver, spleen and lung. The results of the recovery for each of the investigated tissues at day 90 after single and repeated dosing are presented in Figure 9 and Figure 10 for male and female animals respectively. In general, also liver, spleen and lung tissue showed the highest tissue distribution.

An overview of the total recovery (amount titanium in sampled tissue expressed as % of the total administered dose) after single and repeated dosing with NM-100, NM-102, NM-103 and NM-104 for both males and females is shown in Table 3. Total recovery at 24 hours after single administration ranged from 64-95% and 59-108% for male and female animals respectively. NM-103 (females) and NM-104 (males) showed the highest total recovery (108% and 95%, respectively) at this time point after single dosing. Total recovery as measured at 24 hours after the last administration in the repeated exposure groups ranged from 66-95% and 65-88% for male and female animals, respectively. Also NM-103 (male: 95%, female: 88%) and NM-104 (female: 88%) showed highest total recovery at day 90 after repeated dosing.

**Table 3:** Recovery of titanium for male and female rats in the summed investigated organs* as percentage of dose administered after single or repeated dosing

		Day 2/6**	**Day 14**	**Day 30**	**Day 90**
**Single**					
NM-100	Male	75 ± 3	nd	nd	55 ± 3
	Female	72 ± 8	nd	nd	57 ± 1
NM-102	Male	64 ± 4	nd	nd	52 ± 5
	Female	59 ± 6	nd	nd	53 ± 1
NM-103	Male	86 ± 17	nd	nd	91 ± 12
	Female	108 ± 8	nd	nd	103 ± 12
NM-104	Male	95 ± 18	nd	nd	109 ± 7
	Female	89 ± 5	nd	nd	100 ± 4
**Repeated**					
NM-100	Male	71 ± 8	69 ± 4	70 ± 4	59 ± 5
	Female	73 ± 6	nd	nd	61 ± 7
NM-102	Male	66 ± 14	69 ± 6	70 ± 3	54 ± 1
	Female	65 ± 8	nd	nd	55 ± 1
NM-103	Male	95 ± 6	110 ± 21	96 ± 19	72 ± 13
	Female	88 ± 7	nd	nd	84 ± 3
NM-104	Male	75 ± 5	99 ± 3	81 ± 10	77 ± 6
	Female	88 ± 10	nd	nd	86 ± 4

*liver, spleen, kidney, lung, heart, brain, thymus, blood, testes/ovaries.

**Day 2 or day 6 corresponds to single or repeated dosing respectively.

nd: not determined.

Both after single and repeated titanium dioxide IV administration (NM-100, NM-102, NM-103, NM-104), titanium levels in faeces (collected at days 2, 6, 14, 30 or 90 if possible), obtained at the same time as the tissues were collected, were similar to those observed in vehicle control animals (data not shown). In addition, no increase in titanium levels in urine (collected at days 2, 6, 14, 30 or 90 if possible) was observed after repeated exposure in NM-100 and NM-102 exposed animals (no analysis performed in urine of NM-103 and NM-104 exposed animals). This is in agreement with the negligible elimination.

**Figure 11:**
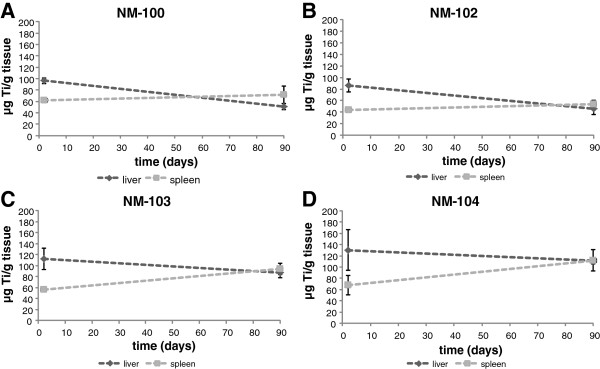
***IV study: *** **Distribution of Ti to the liver and spleen (μg Ti/g organ) after single IV dosing with NM-100 (A), NM-102 (B), NM-103 (C), and NM-104 (D) in male rats.**

**Figure 12:**
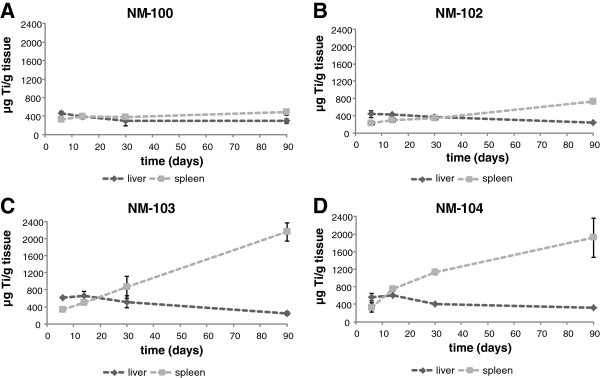
***IV study: *** **Distribution of Ti to the liver and spleen (μg Ti/g organ) after repeated IV dosing with NM-100 (A), NM-102 (B), NM-103 (C), and NM-104 (D) in male rats.**

For both the single as well as the repeated IV exposure, the potential elimination of titanium was measured up to 90 days post-exposure. During this post-exposure period, some decline was observed (mainly for NM-100 and NM-102) with a maximum decrease of 20% (NM-100, males) (Table 3), which corresponds to a maximum relative decrease of 26% (day 90 vs. day 2 or 6). For liver and spleen, these results are shown in Figure 11 (single exposure) and Figure 12 (repeated exposure), respectively. Liver titanium levels showed a decrease during 90 days post-exposure for all titanium dioxide particles together with increased spleen titanium levels. At day 90 post-exposure, titanium levels in spleen were higher as compared to liver titanium levels (expressed as μg/g tissue). This indicates some redistribution between liver and spleen.

Further, data of female animals (titanium levels expressed as μg/g tissue) are presented separately as Additional file 1: Tables S1-S8. In general, female animals showed a similar kinetic profile as male animals. The major difference between male and female animals was the disappearance of Ti in the reproductive organs, which was slower in female animals.

Table 4 presents the results of the kinetic analysis. The results show very long elimination half-lifes for each of the investigated tissues. Further, some differences in Cmax and AUClast are observed between materials, with in general NM-103 and NM-104 showing the highest values.

**Table 4:** **Basic pharmacokinetic parameters of titanium dioxide in different organs after 5 daily administrations of TiO**_**2**_**by the IV route**

**NM**	**Organ**	**β**	**T**_**1/2el**_	**C**_**t**_	**AUClast**
		**(/d)**	**(d)**	(μg/g)*	(μg d/g)**
NM-100	liver	0.0028	248	522.3 ± 23.34 (t = 6)	32790 ± 2306
NM-102	liver	0.0073	95	485.3 ± 62.13 (t = 6)	34682 ± 1195
NM-103	liver	0.0129	54	727.3 ± 64.72 (t = 14)	45919 ± 3684
NM-104	liver	0.0250	28	694.0 ± 10.82 (t = 14)	43491 ± 855
NM-100	kidney	0.0058	120	1.29 ± 0.09 (t = 6)	41 ± 4
NM-102	kidney	0.0047	146	1.45 ± 0.06 (t = 6)	69 ± 2
NM-103	kidney	0.0140	49	3.69 ± 0.19 (t = 6)	190 ± 19
NM-104	kidney	0.0013	531	2.46 ± 0.59 (t = 6)	178 ± 13
NM-100	lung	0.0153	45	54.67 ± 10.27 (t = 90)	4360 ± 381
NM-102	lung	0.0091	76	120.00 ± 19.22 (t = 14)	8733 ± 647
NM-103	lung	0.0072	96	130.67 ± 4.41 (t = 6)	7817 ± 672
NM-104	lung	nc	nc	55.02 ± 5.20 (t = 90)	3791 ± 187
NM-100	spleen	0.0011	650	536.01 ± 37.53 (t = 90)	41175 ± 1437
NM-102	spleen	nc	nc	827.33 ± 33.88 (t = 90)	45909 ± 1241
NM-103	spleen	nc	nc	1833.67 ± 578.34 (t = 90)	102710 ± 18670
NM-104	spleen	nc	nc	2221.667 ± 309.47 (t = 90)	130624 ± 9527
NM-100	heart	0.0048	146	0.81 ± 0.09 (t = 6)	39 ± 1
NM-102	heart	0.0050	140	0.81 ± 0.13 (t = 14)	48 ± 3
NM-103	heart	0.0029	237	1.35 ± 0.06 (t = 6)	85 ± 14
NM-104	heart	nc	nc	1.50 ± 0.26 (t = 90)	78 ± 10

β = apparent elimination rate, T_1/2el_: apparent elimination half-life, Ct: highest concentration at time point t (day), AUClast: area under curve between the between 1^st^ measured concentration and the last measured concentration, nc: not calculated.

*P < 0.05 NM-100/NM-102 vs NM-103/NM-104 (for all tissues).

**P < 0.05 NM-100 vs NM-103/NM-104 (for all tissues).

## Discussion

The present study describes the kinetics of titanium dioxide nanoparticles in rats. Animals were exposed via either the oral or the IV route. As exposure via food-products might contribute significantly to the total exposure of titanium dioxide nanoparticles, evaluation of the absorption from the oral route is relevant for human risk assessment. The IV route was included to obtain insight in the blood and tissue distribution profile under conditions of full systemic bioavailability, and to gain insight in the elimination over a prolonged period of time (90 days).

### Oral absorption (oral study)

The dose levels used in our oral study (i.e. 2.3 mg TiO_2_/animal/day for five days; 6.8-8.5 mg TiO_2_/kg bw/day for male animals and 10.9-12.0 mg TiO_2_/kg bw/day for female animals) are much lower and more realistic than doses used by others in toxicity or kinetic studies, as these typically applied doses greater than 100 mg/kg bw/day and even as high as several thousands of milligrams TiO_2_/kg bw/day [15,30]. The consumption of titanium dioxide for the UK population was reported to be on average 2–3 mg/kg bw/day for children under the age of 10 years and approximately 1 mg/kg bw/day for other consumer age groups [2]. For USA, these numbers were 1–2 mg/kg bw/day and 0.2-0.7 mg/kg bw/day respectively [2]. The dose levels used in our oral study can thus be considered within a realistic and relevant frame for the human situation.

Our data show that after repeated oral exposure (overall dose of 11.5 mg TiO_2_) titanium levels were near or below the detection limit in liver and spleen, indicating a very low absorption. Because of the realistic dose levels used in the present study, a quantitative value for oral absorption is difficult to determine due to the many measurement points below the LOD. Only in two out of 30 liver/spleen samples of exposed animals (NM-102 and NM-103 liver) was the Ti level at or above the LOD. In contrast all MLN samples (controls and exposed) contained Ti amounts above LOD (Figure 3). Only a small increase in Ti content was observed, because the background levels in MLN were 2–3 times the LOD. MLN from control rats contained 0.14 μg Ti whereas the highest Ti average was 0.36 μg and was located in MLN from NM-104 exposed rats. This gives an increase of 0.226 μg Ti in MLN or 0.003% of the 6895 μg Ti exposure in the dose.

The total recovery of dosed Ti in all tested organs (expressed as % of the total dose) was estimated to be approximately 0.02%. This was based on calculations using different scenarios (i.e. using LOD or half the LOD for the non-detects; correcting the tissue levels for background levels; using only the positive liver titanium levels). Not all tissues were included as the oral study focused on liver, spleen and mesenteric lymph nodes as target tissues, which seems appropriate based on the results of the IV study. The data of our oral study indicate that some minor absorption may occur in the GI tract after oral exposure, be it to a very limited extent. However, due to the very limited elimination of the nanoparticles even a low absorption should be considered in risk assessment.

Recently, Tassinari *et al*. (2014) [31] evaluated, in addition to the reproductive and endocrine effects of nano-sized titanium dioxide particles (commercially available, anatase, primary size < 25 nm, BET surface area 45–55 m^2^/g), Ti levels in tissues as thyroid, ovaries and spleen after short-term exposure [31]. Rats were orally exposed for 5 days to 0, 1 or 2 mg/kg bw/day titanium dioxide nanoparticles (doses which are even lower than the doses as applied in our study). A significant increase in titanium levels in spleen (0.046 ± 0.008 μg/g) was observed at the high dose group, though the difference with the control animals was small. These data are in the same range as incidentally observed in our study. Single particle-ICP-MS analysis of the target tissue spleen revealed the presence of both titanium dioxide nanoparticles and their aggregates [31]. The kinetics of nano-sized titanium dioxide in rats after repeated 13-weeks oral exposure were studied by Cho *et al*. [30]. Titanium dioxide particles (commercially available, primary particle size of 26.4 ± 6.1 nm as measured with SEM) were administered to rats in doses of approximately 250, 500 and 1000 mg/kg bw/day, 7 days/week for 13 weeks. Titanium-levels were analyzed using ICP-MS with a reported limit of detection of 0.1-1 ng/L. No clear dose-related increases in titanium levels in liver, spleen, kidney and brain could be observed, indicating very low systemic bioavailability [30]. At high dose levels such as several hundreds to one thousand milligram/kg bw/day, absorption of titanium dioxide might be reduced due to agglomeration/aggregation of the particles in the gastrointestinal tract. For silica particles, it was recently hypothesized that gel-formation occurs, especially at higher particle concentrations, under conditions of a relatively high pH and salt concentration, like in the intestine [32]. This gelation is hypothesized to lead to a decrease in oral absorption with increasing dose. In contrast, Wang *et al*. showed absorption of titanium dioxide nanoparticles after a single high dose oral exposure in mice [15]. They used a very high and quite unrealistic single dose of 5000 mg/kg bw (approximately 100 times higher than the cumulative dose used in our study). Primary particle size of the titanium dioxide particles included in this study was 25, 80 and 155 nm. After a single oral dose, titanium could be detected in liver, spleen, kidney, lung, brain and red blood cells. They identified liver as the main target tissue, where highest uptake (by far) was shown for the 80 nm particle (approximately 4 μg/g; a concentration about 100 times higher than our LOD) [15].

### Tissue distribution (IV study)

Based on the results of our IV study, liver, spleen and lungs were identified as primary target tissues for titanium dioxide nanomaterials. Highest levels were observed in the liver, but redistribution of the liver to the spleen was observed over the 90 day post-exposure period. A similar kinetic profile after 24 hours for titanium dioxide nanoparticles in rats was observed recently [17,22]. In these studies, a single IV administration of titanium dioxide nanoparticles (5 mg/kg bw) resulted in highest levels (in descending order) in liver, spleen, lung and kidney as well [17,22]. After IV administration, titanium levels in blood rapidly declined (both after single as well as repeated administration). These results indicate a fast distribution of titanium dioxide to the various tissues.

The tissue distribution profiles of the four IV tested nanomaterials with different sizes and crystalline forms (NM-100, NM-102, NM-103, NM-104) are in general rather similar, although some differences were observed.

Titanium was detected in all investigated tissues in the present study, i.e. blood, liver, spleen, kidney, lung, heart, brain, thymus and reproductive organs. The total recovery was mainly below 100%. An explanation might be that not all tissues were investigated. Skin and muscle were not included as they were inconsequently measured or an initial experiment showed that the levels were close to the detection limit (data not shown). Bone marrow Ti levels could not be measured due to the small sample size. Based on the relatively constant tissue and low blood levels (after the initial rapid decline), it seems rather unlikely that the incomplete recovery can be explained by elimination processes. This is in agreement with the negligible amount of Ti found in excreta (data not shown).

No large differences in distribution between male and female animals were observed. The major difference between male and female animals was the disappearance of Ti in the reproductive organs. In male animals Ti was not detectable in the testes 30 days after administration, whereas at day 90 Ti was still detectable in the female ovaries. This difference can be explained by the differences in blood supply to the testes and ovaries. On day 6 a similar very low percentage of the dose was distributed to both testes and ovaries.

### Elimination (IV study)

Both after single and repeated IV exposure, blood titanium levels in blood decreased rapidly during the first minutes after which the titanium levels slowly decreased and approached the limit of detection at 24 hours post-dose, similar to what was found earlier for silver nanoparticles in blood by Lankveld *et al*. [8]. In the nanosilver study the sampling period ranged to 17 days instead of 90 days as was the case in our study. During the period of 12 days after exposure, Lankveld *et al.* found a gradual decrease in organ silver levels but silver was still present in several organs including liver, lungs, spleen and kidney [8]. In our study, the total titanium levels slowly, but definitely, reduced up to Day 90, although such a decrease is not obvious from the data at time point day 14 (i.e. 9 days after exposure). Based on these data it can be concluded that elimination of total TiO_2_ has a long half-life, which was also shown by the results of the kinetic analysis presenting half-life in days for the various organs (for example for liver as the main target organ: 28–248 days). In contrast to the titanium dioxide particles, as described here, for NM-105 (an anatase rutile mixture) a major decline in various organ levels was noted [33]. In the present study, some reduction in recovery was observed up to Day 90 for the pigment-sized (NM-100) and one of the nano-sized (NM-102) titanium dioxide particles, with a maximum relative decrease (day 90 vs. day 2 or 6) of 26% (NM-100, males).

Redistribution of titanium from the liver to the spleen was observed between Day 2/Day 6 and Day 90, whereas redistribution to remaining tissues was not identifiable. Release of particles from liver and possibly other organs may be responsible for the increase in spleen levels. The data show that at the long run TiO_2_ particles will accumulate in spleen. The spleen Ti concentration rises during the entire exposure, including the 90 day post exposure period.

In addition, titanium levels as measured in the faeces of IV-treated (single and repeated) animals revealed no clear differences between titanium dioxide-exposed animals and vehicle-treated controls (data not shown). Further, no increase in titanium levels in urine was observed. This further confirms the lack of elimination of the titanium dioxide nanoparticles.

The expected accumulation with daily exposure as a consequence of the negligible elimination might indicate a potential concern for human health risk. Even with very low uptake from the gastrointestinal tract human daily oral exposure can be expected to give rise to a very low but steady increase in titanium levels in tissues in time. Weir *et al*. estimated the exposure to pigment grade TiO_2_ via food in the order of 1 mg/kg bw per day [2]. The nanofraction in these pigmentary TiO_2_ (E171) is estimated to be 10-36% of the number of particles [2,4]. So, the aspects of oral uptake, limited elimination and anticipated accumulation in man should be further investigated in the risk assessment of nanosized TiO_2_.

### Differences between titanium dioxide particles tested

The present study is the first to evaluate the kinetics of different titanium dioxide nanoparticles after both single as well as repeated oral and intravenous administration in rats. The titanium dioxide nanoparticles as used in this study differed with respect to particle size, with NM-100 being the largest particle (nominally 200–220 nm vs. 7–10 nm for NM-101, 15–25 nm for NM-102 and, 20 nm for NM-103 and NM-104). However it should be realized that in the nanomaterial dispersions used, the TiO_2_ nanoparticles are present as either agglomerates (loosely bound particles) or aggregates (more or less fixed particles). Only minor differences in kinetic profile, both after single and repeated exposure, were observed in the present study, which may be linked to differences in size, hydrophobicity, crystalline form or just animal or random variation. For gold nanoparticles we previously observed a difference in tissue distribution depending on size, the smaller nanoparticles showing a more wide spread tissue distribution [10].

In the present study, all particles showed a rapid distribution to the organs from the systemic circulation. The total recovery of NM-103 and NM-104 was higher than NM-100 and NM-102, which might indicate a small difference in tissue distribution. Some indications were noted for a difference in recovery between anatase (NM-102) and rutile nano-TiO_2_ (NM-103 and NM-104). However, these crystalline forms were not compared in one single experiment, making interpretation of these differences difficult.

Further, elimination from the studied organs was very slow, with no difference between the titanium dioxide particles observed. It should be noted that in the present study we did not systematically change a specific physicochemical characteristic of the selected titanium dioxide particles. Such an approach could facilitate the detection of differences in kinetic behavior in relation to such a characteristic. In the present study commercially available titanium materials were used, which is relevant from a risk assessment point of view. At the moment it is not possible to combine these aspects.

## Conclusions

The titanium dioxide nanoparticles evaluated in this study showed very limited bioavailability after oral exposure, though there was evidence that absorption is possible in the gastrointestinal tract as increased levels of titanium could be detected in some livers and mesenteric lymph nodes in exposed animals compared to control animals. It can be further concluded that after systemic exposure, the titanium dioxide nanoparticles mainly distributed to the liver, spleen and lung. Though the studied particles differed to some extent with respect to primary particle size, some but no major differences in tissue distribution were observed. Repeated exposure resulted in increased but dose-proportional tissue levels. Tissue redistribution was observed from the liver to the spleen over a period of 90 days after exposure, with an increase rather than a decrease in Ti spleen concentration during the 90 day post-exposure period. Further, elimination was very slow with only small amounts titanium dioxide eliminated in up to 90 days post-exposure. Elimination was most pronounced for the pigment-sized (NM-100) and one of the nano-sized titanium particles (NM-102).

Overall, the results of the present study indicates very low oral bioavailability and slow tissue elimination. Limited uptake in combination with slow elimination might result in the long run, particularly with daily exposure, in potential tissue accumulation. This is especially important for the human risk assessment of these materials.

## Material and methods

### Animals

#### Oral study

Male (N = 30) and female (N = 6) Wistar rats (CRL:WI (WU)) were obtained from Charles River, Germany. They were delivered as 7 weeks old and were randomly divided into groups of two males or three females. They were housed in polycarbonate cages with a bottom area of 905 cm^2^ and a height of 21.5 cm, with bedding, feed, enrichment, light and temperature/humidity as described previously [34]. The cages were sanitized twice weekly. The rats were barrier maintained and were allowed to acclimatize for 2 weeks before they entered the experimental protocol. All rats were 9 weeks old at the time of the experiment. The experiments were conducted at NRCWE (Copenhagen, Denmark). All animal procedures followed the guidelines for the care and handling of laboratory animals established by the Danish government, and the local animal welfare body as well the National Animal Experiment Inspectorate under the Ministry of Justice, approved the study.

#### IV study

Male and female Wistar rats (CRL:WI (WU)) were obtained from Charles River, Germany. Exposure started after 1–2 weeks of acclimatization. All rats were 9–10 weeks old at the start of the experiment. Rats were randomly divided into groups of 1-3/cage. Animals were bred under specific pathogen-free (SPF) conditions and barrier maintained under controlled conditions of temperature, humidity and light during the experiments. Standard feeding chow diet and water were allowed *ad libitum*. The experiments were conducted at the National Institute for Public Health and the Environment (RIVM, Bilthoven, The Netherlands) according to a protocol approved by the local Ethics Committee for Animal Experiments and according to European and national laws regulating the use of laboratory animals.

### Particles

Five different titanium dioxides (TiO_2_) were used in this study (i.e. NM-100, NM-101, NM-102, NM-103, NM-104), where the material numbers refer to JRC nanomaterials repository [35]. See Table 5 for information on the particles. Additional characterization of the test materials is described below.

**Table 5:** Characteristics* of titanium dioxide nanomaterials

	**NM-100**	**NM-101**	**NM-102**	**NM-103**	**NM-104**
***Use***	Pigment (multiple)	Photocatalyst	Photocatalyst	Cosmetics	Cosmetics
***Crystal form***	anatase	anatase	anatase	rutile (hydrophobic)	rutile (hydrophilic)
***Nominal particle size (nm)***	200 - 220	7 - 10	15 - 25	20	20
***Mean particle size (nm)***	267	38	132	186	67
***Primary particle or crystal size (nm)***	42-90	6	20	20	20
***Specific surface area (m***^***2***^ ***/g)***	10	320	90	60	60

*Particle characterization as described in [33,35].

### Characterisation of test material

Particle characterisation of the powder was performed as described recently [33].

In addition, the size distribution was determined in the dispersions used in the oral and IV studies so as to gain insight in the quality of the dispersions used and to get information on the exposure conditions.

#### Oral study

The hydrodynamic particle number size distribution of the TiO_2_ particles (NM-101, NM-102, NM-103, NM-104) in the exposure liquids were analysed by photon correlation spectroscopy using a Dynamic laser scattering (DLS) Zetasizer nano ZS (Malvern Inc., UK). DLS for each material were performed on two separate days each of 6 measurements. The number distributions were calculated by the DTS software using the viscosity for H_2_O (0.6864), temperature of 25°C and material refractive (Ri) and absorption indices (Rs) for TiO_2_ (Anatase: Ri 2.49; Rs 0.10 and rutile: Ri 2.90; Rs 0.10). Vehicle controls were analysed using both settings. TiO_2_ containing and vehicle samples were analysed using a laser attenuation factor of 2 and 7 and a measure position of 0.45 and 4.65 mm, respectively. All samples were analysed within 30 min after sonication, i.e. within the same time frame as the oral exposures occurred.

#### IV study

The size distribution of the TiO_2_ particles (NM-100, NM-102, NM-103 and NM-104) were determined directly after preparing the suspensions for IV administration in at least 3 separate measurements using tracking analysis of Brownian motion with a laser illuminated microscopical technique (LM20, NanoSight Ltd, UK).

### Test material preparation (oral and IV study)

Particle suspensions were made fresh every day according a standardized protocol [to be published elsewhere]. Most of our practices for ultrasonic dispersion of nanoparticle suspension were in agreement with recommendations by Taurozzi *et al.*[36]. A 2.56 mg/mL stock suspension was prepared by pre-wetting the TiO_2_ powder in 96% ethanol (resulting in a final concentration of 0.5 vol% ethanol) followed by dispersion in 0.05 wt% Rat Serum Albumin (RSA) (Sigma #A6272) in ultrapure water. Probe sonication of the suspensions was performed on ice (Branson Sonifier S-450D, Branson Ultrasonics Corp., Danbury, CT, USA, equipped with a disruptor horn Model number: 101-147-037) for 16 minutes (400 W system and used with a 10% amplitude). The stock suspensions were diluted (9:1 v/v) with 10x concentrated phosphate buffer pH 7.4 (702 mg NaH_2_PO_4_ x 2H_2_O, 4155 mg Na_2_HPO_4_ x 7H_2_O, dissolved in 1 L) resulting in an exposure suspension with a concentration of 2.304 mg/mL TiO_2_. Vehicle control suspensions were also sonicated and diluted according to above description. All suspensions were used immediately. The TiO_2_ nanomaterials were evaluated for the presence of endotoxin. All preparations investigated showed endotoxin levels below 20 IU. Absence of LPS (endotoxin) was further evaluated and confirmed by fatty acid analysis.

### Experimental protocol

Rats were treated orally or intravenously according to the schedule as presented in Table 6. The kinetics of NM-100 was studied for the IV route only, whereas the kinetics of NM-101 was studied for the oral route only. The kinetics of NM-102, NM-103 and NM-104 was studied after both oral and IV administration.

**Table 6:** Schematic overview of the design of the toxicokinetic study for determination of tissue distribution of titaniumdioxide nanomaterials

**Material**	**Daily dose (mg TiO**_**2**_**/animal)**	**# daily doses**	**Route**	**Autopsy**				
				**Day 2**	**Day 6**	**Day 14**	**Day 30**	**Day 90**
NM*	2.3	1	IV	3 M + 3 F	-	-	-	3 M + 3 F
NM*	2.3	5	IV	-	3 M + 3 F	3 M	3 M	3 M + 3 F
NM*	2.3	1	Oral	3 M				
NM*	2.3	5	Oral	-	3 M + 3 F**	-	-	-
Vehicle	-	1 or 5	IV	2 M + 1 F	2 M + 1 F	-	-	2 M + 1 F
Vehicle	-	1 or 5	Oral	2 M + 1 F	2 M + 1 F	-	-	2 M + 1 F

*NM is NM-100, NM-102, NM-103 or NM-104 for the IV route and NM-101, NM-102, NM-103, or NM-104 for the oral route.

**female animals for NM-101 only.

#### Oral study

The rats were dosed by gavage with 1 mL of the vehicle or TiO_2_-suspension according to international accepted guidelines [37,38]. The rats were not sedated or deprived of feed before or after administration of the test substance. The rats were dosed using a straight metal feeding needle with bulbed tip. The length of the inserted needle was from lips to the last rib of the animal ensuring deposition in the stomach. Once the needle was in place, the animal was observed to be properly breathing before dosing 1 mL of the TiO_2_ suspensions. No problems related to the exposure (e.g. reflux or vomiting) were observed. Animals were dosed orally either once (3 males per group, 4 TiO_2_ nanomaterials and controls) or during five consecutive days (3 males per group, 4 TiO_2_ nanomaterials and controls; in addition 3 females per group for NM-101 and controls). The rats were sacrificed and tissue sampling was done 24 h after the last exposure (Day 2 or Day 6).

#### IV study

The rats received a single or repeated dose of 1 mL (on 5 consecutive days) via injection in the tail vein according to international accepted guidelines [37,38]. This volume was frequently used in previous papers without any problems [8-10]. Blood and tissue samples were collected at Day 2 and Day 90 for the single IV administration (3 male and 3 female animals per group), and at Day 6 (i.e. the first day after the last repeated exposure), 14, 30 and 90 after the repeated IV administration (3 male and 3 female animals per group, except for Day 14 and Day 30 which included 3 male animals only). Additional blood samples were collected via orbita puncture at Day 1 (single dose) and Day 5 (repeated doses) at 5, 10, 20 and 30 minutes, and 1, 2, 4, 8, and 24 hours after dosing in order to evaluate the elimination of Ti from the blood after the IV administration. Control rats (vehicle treated) were included (2 male and 1 female animals per group).

### Doses administered

#### Oral study

The doses applied were the maximum concentration that resulted in a stable TiO_2_ nanomaterial dispersion [to be published elsewhere]. The doses are also considered to be in the realistic human exposure range [2,3].

For the oral route a dispersion of the various TiO_2_ nanomaterials was used containing 2.3 mg TiO_2_ per mL. The single dose groups received one single dose of 2.3 mg/rat corresponding to 6.8-8.6 mg/kg bw for male rats depending on the actual weight of the rats. The repeated dose groups received five consecutive daily (Day 1–5) administrations of 2.3 mg TiO_2_ in one mL per rat resulting in a cumulative dose range of 34.1-42.4 mg/kg bw for male orally treated rats and 54.5-59.9 mg/kg bw for female rats.

#### IV study

A dispersion of the various TiO_2_ nanomaterials was used containing 2.3 mg TiO_2_ per mL of which 1 mL was injected intravenously. Preliminary acute toxicity studies (single IV dose of 2.3 mg/rat) indicated that the maximum dose was well tolerated. The single IV treated rats received a dose of 8.4-9.8 mg/kg bw and 12.4-14.1 mg/kg bw for male and female rats respectively. The repeated IV treated rats received a cumulative dose that ranged from 42.3-49.4 mg/kg bw and 61.2-71.9 mg/kg bw for male and female rats, respectively. Thus the actual dose in mg/kg bw depended on the weight of the rats.

### Blood- and tissue sampling

#### Oral study

Twenty four hours after the last oral dosing the rats were anaesthetised subcutaneously with 0.3 mL/100 g Hypnorm® (fentanyl citrate 0.315 mg/mL and fluanisone 10 mg/mL; Janssen Pharmaceuticals)/Dormicum® (Midazolam 5 mg/mL; Roche)/water mixture (1:1:2). The rats were killed by exsanguination via the heart and the following tissues were collected: spleen, liver and mesenteria with mesenteric lymph nodes (designated MLN) were selected as primary target tissues. Full wet weight of these tissues was noted. However, the parts of liver immediately surrounding the gastro intestinal (GI) tract were not taken, since this could lead to rupture of the GI tract and to TiO_2_ contamination of tissues. The full liver weight might therefore be slightly underestimated. Additionally, blood (450 μl weighing 0.5 g), kidney, lungs, heart, brain, thymus, muscle, bone/bone marrow, testes or ovaria, and skin were collected. All tissues were cooled on dry ice and stored at -20°C.

#### IV study

After IV administration the rats were anaesthetized by inhalation of isoflurane (Isoflu®, AST Pharma, Oudewater, The Netherlands) in oxygen and subsequently euthanized by exsanguination via the abdominal aorta. Next to blood (0.5-1.0 mL) the following tissues were collected: liver, spleen, kidneys, lungs, heart, brain, thymus, skin, and testes or ovaria. Urine and feces where collected when feasible, i.e. when present in either bladder or colon/rectum. In addition, lymph nodes (mesenteric and popliteal), bone including bone marrow (femur), and muscle were collected as well. Blood (100–200 μl) was collected via orbita puncture at day 1 (for single exposure) and day 5 (for repeated exposure) to evaluate Ti elimination from the blood circulation. All tissues and blood were cooled on dry ice and stored at -20°C.

### HR-ICP-MS analysis of the tissues

The tissue distribution of the TiO_2_ nanomaterials administered to the animals was evaluated by determining the Ti content in blood and tissues at various time points after administration by High-Resolution Inductively Coupled Plasma Mass Spectrometry (HR-ICP-MS), as recently developed and described [39]. For the oral study, the livers were homogenized in 50 mL centrifuge tubes for ~30 sec./level 2 using an Ultra Turrax T25 Basic (IKA Labortechnik, Staufen, Germany). Spleens and mesenteria with lymph nodes were homogenized for 6 min in a 2 mL cryotube using a Qiagen Tissue Lyzer at 30 strokes per sec. For the IV study, the tissues were homogenized by manual cutting and stirring. Depending on the weight either the whole tissue or subsamples (about 0.5 - 1 gram) were used for the Ti analysis.

A homogenized tissue sample was weighed (approximately 0.5 g) into 15 mL polypropylene tubes (Sarstedt BV, Etten-Leur, The Netherlands) and 0.5 mL ultrapure water, 1 mL nitric acid conc. (HNO_3_) and 0.75 mL hydrofluoric acid conc. (HF) were added. The tubes were placed on a block heater (type: Stuart SBH200D; supplied by Omnilabo, Breda, The Netherlands) and the mixture was slowly heated to a final temperature of 90°C. The mixture was left for two days at this temperature. Afterwards, ultrapure water was added to a total volume of 15 mL. With the applied procedure, it was possible to achieve a good dissociation of the original nanoparticles as well as digestion of the organic materials.

The Ti measurements were performed at Philips Innovation Services (Eindhoven, The Netherlands) with a HR-ICP-MS (ELEMENT XR, Thermo Fisher Scientific, Breda, The Netherlands), using a set-up with on-line addition of an internal standard. Two Ti isotopes were measured in the medium resolution (MR) mode: while ^47^Ti(MR) was used for the evaluation, ^49^Ti(MR) was used for control. The accurate masses and abundances are: ^47^Ti (46.95176 atm): 7.3%, respectively ^49^Ti (48.94787 atm): 5.5%. The element Ti consists in total of five naturally abundant isotopes. Nevertheless, the other three isotopes cannot be used for quantification by HR-ICPMS due to isobaric and polyatomic interferences. The instrumental operating conditions were as follows: RF power 1225 W, cooling gas flow 16 L/min argon, auxiliary gas flow 0.9 L/min argon, and the sample gas flow 0.99 L/min argon.

The solutions were measured against an external calibration with internal standard correction using ^115^In. As no standard reference materials are available for Ti determination a commercial reference material obtained from Sero AS (Seronorm™ Trace Elements Whole Blood, Sero AS, Billingstad, Norway) was analysed according to the same procedure (L-2 LOT 1003129 contained 18 ± 5 μg/L Ti, and L-3 LOT 1112691 contained 12.8 ± 0.4 μg/L Ti as provided by the supplier). The limit of detection (LOD; as 3x STD, n = 20) is estimated from the results obtained for approximately 0.5 g tissue material from control animals of this study after applying the complete procedure of digestion and measurement with HR-ICPMS. The LOD is 0.05 μg Ti/g tissue and the LOQ 0.15 μg Ti/g tissue. For the tissue series evaluated for Ti content after oral administration (for five consecutive days) the LOD was 0.03 μg Ti/g tissue, and the LOQ 0.09 μg Ti/g tissue.

### Kinetic analysis and statistical analysis

Pharmacokinetic analysis was performed on sparse data by a non-compartmental approach (NCA) using Phoenix WinNonlin 6.3 software (Pharsight Corporation, Mountain View, CA, USA) [40]. The analysis focused on the organ kinetic parameters. Kinetic parameters such as AUC and apparent elimination rate (β) were determined. The AUC represents the ‘area under the curve’ of the organ Ti concentration – time curve, and was calculated using the linear trapezoidal rule. Apparent elimination rate (β) was estimated on the terminal phase of the curve by linear regression of at least 3 points (r^2^ > 0.95). The apparent elimination half-life (T_1/2el_) was calculated by the following equation:

$$T_{1/2\mathrm{el}}=\frac{0.693}{\beta }$$

Standards errors were only estimated for Cmax and AUC.

Statistical analysis was performed using the nonparametric Friedman test followed by a pairwise comparison using Wilcoxon test to locate potential differences between groups (i.e. nanomaterials and tissues). The significance level was 0.05. All statistical analyses were performed using SYSTAT 13 (Systat Software Inc, Chicago, USA).

### Data evaluation

Data of male and female animals were evaluated separately as the difference in dose levels between these groups (as a result of variation in body weight between male and female animals) was considered too large. Further, the number of sampling periods differed between male and female animals. However, data within the male animal groups or within the female animal groups were analysed together and the titanium levels (expressed per tissue or g tissue) were not corrected for differences in body weight and thus in dose (mg/kg bw) within these groups. This was considered acceptable, as the intra-group variation (max. 8%; as a result of intra-group differences in body weight) was not expected to exceed the standard random animal variation.

In groups containing both rats with a Ti level above and below the LOD, a simulant value was used for measurements below the LOD for estimation of the mean Ti for all animals investigated. By using a simulant value the information on animals with a measurement below the LOD was included in the estimation of the Ti concentration per gram organ for the group of animals. The non-detect samples were assumed to contain Ti concentrations equal to half the LOD, in order to be able to use the information on these tissues in the calculations. A value of 0 was considered to be too low a value introducing a bias to a low mean value, whereas using the LOD itself was considered to result in a bias towards an overestimation of the tissue Ti content.

For estimating the recovery, the absolute amounts of titanium per tissue were calculated using the corresponding tissue weights for each animal with the individual dose. Second, the amounts of titanium dioxide dosed via single exposure (2.3 mg TiO_2_) or repeated exposure (11.5 mg TiO_2_) were converted into amounts of titanium (1379 and 6895 μg Ti respectively). This was done by multiplying the amounts of titanium dioxide with the ratio of the molecular masses of “titanium” and “titanium dioxide” (i.e., ~0.60). Finally, the absolute amounts of titanium in tissue were expressed as percentage of the total (single or cumulative) administered dose.

## Competing interests

The authors declare that they have no competing interests.

## Authors’ contributions

LG analysed and interpreted data, and drafted the manuscript. AGO contributed to the experimental design, interpreted data, contributed to the coordination and drafting of the manuscript. PK coordinated the Ti-analysis, contributed to the drafting of the manuscript. NRJ contributed to the experimental design, interpreted the data, contributed to the drafting of the manuscript. HW contributed to the experimental design and drafting of the manuscript. ML conducted the kinetic and statistical analyses. HWV contributed to the experimental design, and supervised and coordinated part of the animal experiments. EFAB analysed and interpreted the data, contributed to the drafting of the manuscript. WHJ contributed to the experimental design, interpreted the data, contributed to the drafting of the manuscript and was responsible for overall coordination of the studies. All authors read and approved the final manuscript.

## Supplementary Material

Additional file 1 Supplementary material: Overview of titanium-levels of female animals. Click here for file
