# Supplementary material for: Tissue distribution and elimination after oral and intravenous administration of different titanium dioxide nanoparticles in rats
Source: Part Fibre Toxicol. 2014 Jul 3;11:30. doi: 10.1186/1743-8977-11-30 (PMC4105399; doi:10.1186/1743-8977-11-30)
Supplement: Additional file 1 — Supplementary material: Overview of titanium-levels of female animals. [file 1743-8977-11-30-S1.docx]

**SUPPLEMENTARY FILE**

**Table S1**

Ti tissue distribution (µg/g tissue) in female rats after single and repeated intravenous TiO_2_ (NM-100) administration.

| **NM-100** | **Single exposure** | | **Repeated exposure** | |
| --- | --- | --- | --- | --- |
|  | **Day 2** | **Day 90** | **Day 6** | **Day 90** |
| Liver | 175 ± 53 | 85 ± 8 | 576 ± 22 (2) | 470 ± 133 |
| Spleen | 95 ± 16 | 122 ± 25 | 414 ± 18 | 827 ± 8 (2) |
| Kidney | 0.35 ± 0.09 | 0.06 ± 0.01 | 0.98 ± 0.12 | 0.27 ± 0.07 |
| Lung | 13.3 ± 5.1 | 12 ± 9 | 65 ± 9 | 55 ± 26 |
| Heart | 0.20 ± 0.03 | 0.13 ± 0.05 | 0.66 ± 0.18 | 0.67 ± 0.13 (2) |
| Brain | 0.05 ± 0.00 | < 0.05 (2) | 0.06 ± 0.01 | 0.06 ± 0.01 (2) |
| Thymus | <0.05 | 0.17 ± 0.18 | 0.22 ± 0.24 | 1.24 ± 0.95 |
| Ovaries | 0.08 ± 0.03 | 0.10 ± 0.06 | 0.41 ± 0.03 | 0.26 ± 0.19 |
| Skin | 0.09 ± 0.06 | nd | 0.13 ± 0.03 | nd |
| Blood | 0.10 ± 0.07 | < 0.05 | 0.15 ± 0.05 | <0.05 |

Data expressed as mean and standard deviation in µg/g tissue, number of animals investigated n=3 unless otherwise indicated within brackets, nd= not determined

**Table S2**

Ti tissue distribution (µg/g tissue) in female rats after single and repeated intravenous TiO_2_ (NM-102) administration.

| **NM-102** | **Single exposure** | | **Repeated exposure** | |
| --- | --- | --- | --- | --- |
|  | **Day 2** | **Day 90** | **Day 6** | **Day 90** |
| Liver | 115 ± 13 | 85 ± 9 | 640 ± 40 | 402 ± 48 |
| Spleen | 47 ± 11 | 97 ± 43 | 271 ± 17 | 730 ± 96 |
| Kidney | 0.57 ± 0.43 | 0.09 ± 0.03 | 1.29 ± 0.23 | 0.62 ± 0.23 |
| Lung | 25 ± 23 | 19 ± 5 | 105 ± 9 | 128 ± 31 |
| Heart | 0.15 ± 0.04 (2) | 0.14 ± 0.03 | 0.63 ± 0.08 | 0.62 ± 0.06 |
| Brain | <0.05 | <0.05 (2) | 0.11 ± 0.09 | 0.06 ± 0.03 (2) |
| Thymus | <0.05 | 0.17 ± 0.08 | 0.25 ± 0.19 | 0.77 ± 0.09 (2) |
| Ovaries | 0.12 ± 0.14 | <0.05 (2) | 0.29 ± 0.04 | 0.25 ± 0.11 |
| Skin | 0.06 ± 0.04 | nd | 0.12 ± 0.03 | nd |
| Blood | <0.05 | <0.05 | 0.10 ± 0.04 | <0.05 |

Data expressed as mean and standard deviation in µg/g tissue, number of animals investigated n=3 unless otherwise indicated within brackets, nd= not determined

**Table S3**

Ti tissue distribution (µg/g tissue) in female rats after single and repeated intravenous TiO_2_ (NM-103) administration.

| **NM-103** | **Single exposure** | | **Repeated exposure** | |
| --- | --- | --- | --- | --- |
|  | **Day 2** | **Day 90** | **Day 6** | **Day 90** |
| Liver | 150 ± 1 (2) | 178 ± 28 | 939 ± 294 | 663 ± 51 |
| Spleen | 87 ± 4 (2) | 152 ± 13 | 359 ± 23 | 1949 ± 376 |
| Kidney | 0.86 ± 0.21 (2) | 0.39 ± 0.20 | 3.20 ± 0.46 | 1.40 ± 0.17 |
| Lung | 28 ± 8 (2) | 16 ± 4 | 132 ± 36 | 104 ± 8 |
| Heart | 0.40 ± 0.05 (2) | 0.55 ± 0.24 | 1.50 ± 0.35 | 1.15 ± 0.26 |
| Brain | 0.10 ± 0.03 (2) | <0.05 | 0.47 ± 0.28 | 0.10 ± 0.02 |
| Thymus | 0.15 ± 0.05 (2) | 0.83 ± 0.23 | 1.19 ± 0.47 | 1.55 ± 0.49 (2) |
| Ovaries | 0.28 ± 0.01 (2) | 0.54 ± 0.50 | 0.94 ± 0.23 | 4.47 ± 1.43 |
| Skin | nd | nd | nd | nd |
| Blood | <0.05 (2) | nd | 0.08 ± 0.03 | nd |

Data expressed as mean and standard deviation in µg/g tissue, number of animals investigated n=3 unless otherwise indicated within brackets, nd= not determined

**Table S4**

Ti tissue distribution, expressed as µg/g tissue, in female rats after single and repeated intravenous TiO_2_ (NM-104) administration.

| **NM-104** | **Single exposure** | | **Repeated exposure** | |
| --- | --- | --- | --- | --- |
|  | **Day 2** | **Day 90** | **Day 6** | **Day 90** |
| Liver | 198 ± 24 | 166 ± 7 | 968 ± 149 | 620 ± 52 |
| Spleen | 98 ± 17 (2) | 194 ± 28 | 404 ± 7 | 2699 ± 239 |
| Kidney | 0.40 ± 0.00 | 0.22 ± 0.12 | 2.30 ± 0.26 | 1.50 ± 0.10 |
| Lung | 6.3 ± 0.6 | 5.7 ± 1.5 | 53 ± 1 | 57 ± 3 |
| Heart | 0.24 ± 0.03 | 0.09 ± 0.01 | 1.07 ± 0.55 | 1.19 ± 0.28 |
| Brain | <0.05 | <0.05 | 0.12 ± 0.01 | 0.06 ± 0.01 (2) |
| Thymus | 0.07 ± 0.02 | 0.08 ± 0.03 | 0.57 ± 0.22 | 2.10 ± 0.28 (2) |
| Ovaries | 0.24 ± 0.03 | 0.16 ± 0.12 | 1.10 ± 0.17 | 0.55 ± 0.48 |
| Skin | nd | nd | nd | nd |
| Blood | < 0.05 | nd | 0.08 ± 0.03 | nd |

Data expressed as mean and standard deviation in µg/g tissue, number of animals investigated n=3 unless otherwise indicated within brackets, nd= not determined

**Table S5**

Blood kinetics of Ti, expressed as µg/g blood, in female rats after single and repeated intravenous TiO_2_ (NM-100) administration

| **NM-100** |  |  |
| --- | --- | --- |
|  |  |  |
| **Time (h)** | **single** | **repeated** |
| 0.08 | 1.85 ± 0.49 (2) | 0.36 (1) |
| 0.17 | 0.36 ± 0.03 (2) | 0.26 (1) |
| 0.33 | 0.28 ± 0.08 (2) | 0.22 (1) |
| 0.5 | 0.22 ± 0.04 (2) | 0.47 (1) |
| 1 | 0.16 ± 0.06 (2) | 0.21 (1) |
| 2 | 0.18 ± 0.13 (2) | 0.53 (1) |
| 24 | 0.095 ± 0.07 (3) | 0.15 ± 0.05 (3) |

Data expressed as mean and standard deviation in µg/g, within brackets number of animals investigated

**Table S6**

Blood kinetics of Ti, expressed as µg/g blood, in female rats after single and repeated intravenous TiO_2_ (NM-102) administration

| **NM-102** |  |  |
| --- | --- | --- |
|  |  |  |
| **Time (h)** | **single** | **repeated** |
| 0.08 | 1.65 ± 0.49 (2) | 0.13 (1) |
| 0.17 | 0.1 ± 0.01 (2) | 0.14 (1) |
| 0.33 | 0.09 ± 0.04 (2) | 0.19 (1) |
| 0.5 | 0.085 ± 0.01 (2) | 0.2 (1) |
| 1 | 0.085 ± 0.05 (2) | 0.24 (1) |
| 2 | 0.105 ± 0.05 (2) | 0.32 (1) |
| 24 | 0.053 ± 0.05 (3) | 0.1 ± 0.04 (3) |

Data expressed as mean and standard deviation in µg/g, within brackets number of animals investigated

**Table S7**

Blood kinetics of Ti, expressed as µg/g blood, in female rats after single and repeated intravenous TiO_2_ (NM-103) administration

| **NM-103** |  |  |
| --- | --- | --- |
|  |  |  |
| **Time (h)** | **single** | **repeated** |
| 0.08 | 0.17 ± 0.01 (2) | 0.425 ±0.22 (2) |
| 0.17 | 0.0475 ± 0.03 (2) | 0.125 ± 0.06 (2) |
| 0.33 | 0.0425 ± 0.02 (2) | 0.13 ± 0.00 (2) |
| 0.5 | <LOD (2) | nd |
| 1 | <LOD (2) | nd |
| 2 | <LOD (2) | nd |
| 24 | <LOD (2) | 0.08 ± 0.03 (3) |

Data expressed as mean and standard deviation in µg/g, within brackets number of animals investigated, nd= not determined

**Table S8**

Blood kinetics of Ti, expressed as µg/g blood, in female rats after single and repeated intravenous TiO_2_ (NM-104) administration

| **NM-104** |  |  |
| --- | --- | --- |
|  |  |  |
| **Time (h)** | **single** | **repeated** |
| 0.08 | 0.075 ± 0.01 (2) | 0.255 ± 0.02 (2) |
| 0.17 | <LOD (2) | 0.59 ± 0.27 (2) |
| 0.33 | <LOD (2) | 0.305 ± 0.04 (2) |
| 0.5 | <LOD (2) | nd |
| 1 | <LOD (2) | nd |
| 2 | <LOD (2) | nd |
| 24 | <LOD (3) | 0.083 ± 0.03 (3) |

Data expressed as mean and standard deviation in µg/g, within brackets number of animals investigated, nd= not determined
